# Measurement validation of treatment planning for a MR‐Linac

**DOI:** 10.1002/acm2.12651

**Published:** 2019-06-29

**Authors:** Xinfeng Chen, Eric S. Paulson, Ergun Ahunbay, Aydin Sanli, Slade Klawikowski, X. Allen Li

**Affiliations:** ^1^ Department of Radiation Oncology Medical College of Wisconsin Milwaukee WI USA

**Keywords:** beam model verification, electron returning effect, MR‐guided RT, MR‐Linac, treatment planning system

## Abstract

**Purpose:**

The magnetic field can cause a nonnegligible dosimetric effect in an MR‐Linac system. This effect should be accurately accounted for by the beam models in treatment planning systems (TPS). The purpose of the study was to verify the beam model and the entire treatment planning and delivery process for a 1.5 T MR‐Linac based on comprehensive dosimetric measurements and end‐to‐end tests.

**Material and methods:**

Dosimetry measurements and end‐to‐end tests were performed on a preclinical MR‐Linac (Elekta AB) using a multitude of detectors and were compared to the corresponding beam model calculations from the TPS for the MR‐Linac. Measurement devices included ion chambers (IC), diamond detector, radiochromic film, and MR‐compatible ion chamber array and diode array. The dose in inhomogeneous phantom was also verified. The end‐to‐end tests include the generation, delivery, and comparison of 3D and IMRT plan with measurement.

**Results:**

For the depth dose measurements with Farmer IC, micro IC and diamond detector, the absolute difference between most measurement points and beam model calculation beyond the buildup region were <1%, at most 2% for a few measurement points. For the beam profile measurements, the absolute differences were no more than 1% outside the penumbra region and no more than 2.5% inside the penumbra region. Results of end‐to‐end tests demonstrated that three 3D static plans with single 5 × 10 cm^2^ fields (at gantry angle 0°, 90° and 270°) and two IMRT plans successfully passed gamma analysis with clinical criteria. The dose difference in the inhomogeneous phantom between the calculation and measurement was within 1.0%.

**Conclusions:**

Both relative and absolute dosimetry measurements agreed well with the TPS calculation, indicating that the beam model for MR‐Linac properly accounts for the magnetic field effect. The end‐to‐end tests verified the entire treatment planning process.

## 1 INTRODUCTION

Integrated MRI guided radiation therapy (MRgRT) systems are emerging radiation therapy (RT) techniques that combine a MR‐scanner with either a linear accelerator (MR‐Linac)^1^, ^2^, ^3^ or Co‐60 teletherapy system.^4^ The main magnetic field of the MRI is oriented either longitudinal or transverse to the central axis of the radiation beam. Owing to MRI's capability to provide excellent soft‐tissue contrast images and biological/functional information, these systems are expected to enable adaptive RT and provide real‐time, high quality image guidance during delivery with the potential to significantly improve RT outcomes. However, the presence of the magnetic field (ranging from 0.35 to 1.5 T) has nonnegligible dosimetric effects in patients due to the Lorenz deflection effect of the secondary electrons from the primary photon beam. The responses of the radiation dose measurement devices are also affected by the presence of the magnetic field.

The dosimetric effect of a transverse magnetic field (TMF) in phantom and patients has been studied extensively.^5^, ^6^, ^7^, ^8^, ^9^, ^10^, ^11^, ^12^, ^13^, ^14^, ^15^, ^16^ Compared to conventional external beam RT without magnetic field, the magnetic field can generally lead to an altered buildup depth, and a larger, slightly shifted (asymmetric) penumbra arising from tilting of the dose kernel.^5^ The dose deposited at tissue‐air interfaces can increase due to the electron returning effect (ERE),^6^ which can also be affected by the surface orientation where the photon beam enters or exits.^8^ The dosimetric response of QA devices^17^, ^18^, ^19^, ^20^, ^21^, ^22^, ^23^, ^24^, ^25^, ^26^, ^27^ (especially the detectors used for beam calibration) in magnetic fields have also been studied, focusing mainly on the absolute dose response of the detectors. The effect of the magnetic field on the detectors is demonstrated^26^ not only through the trajectory path deflection of the secondary electrons in the medium surrounding and inside the volume of the gas‐filled or solid detector, but also through the change of the intrinsic properties of detectors such as charge carrier versus lattice defect recombination, polarity effect, etc. For the ionization chamber, however, when the field size is sufficiently larger than the detector's diameter, an additional magnetic field and beam quality correction factor^18^ can be applied to the original calibration coefficient, obtained without the magnetic field, to obtain the correct absolute dose calibration in the presence of the magnetic field. The effective point of measurement (EPOM) of the ionization chamber^27^ is shifted not only in the beam direction but also laterally, perpendicular to both the beam and the magnetic field direction. According to O'Brien et al.^27^, the measured lateral shift in the dose distribution was independent of depth and field size from 2 × 2 cm^2^ to 10 × 10 cm^2^. The depth of maximum dose had little dependence on field size in the presence of the magnetic field. The output factors measured at the point of the peak intensity in the cross plane profile are more consistent than those measured at the central axis (CAX).

The MR‐Linac system developed by Elekta AB (Stockholm, Sweden) in cooperation with Philips Healthcare (Best, Netherlands) consists of an Elekta linear accelerator with a nominal 7MV flattening filter‐free photon beam (160‐leaf MLC oriented in fixed superior‐inferior direction), and a Philips 1.5 T integrated wide‐bore MRI scanner. With this system, the magnetic field is oriented transverse to the irradiation field. To match the MR‐Linac, the treatment planning system (TPS) must incorporate the effects of magnetic fields. A prototype TPS (Monaco research version v5.0.19.03, provided by Elekta AB) developed for the MR‐Linac employs a graphics processing unit (GPU)‐based Monte Carlo dose calculation algorithm^28^ and considers a uniform magnetic field with strength and orientation as input.^29^ The main purposes of this study were to: (a) validate the beam models in the TPS by comparing the model predictions with comprehensive dosimetric measurement data from the MR‐Linac and (b) verify the performance of the entire planning and delivery process by carrying out a series of end‐to‐end tests on the MR‐Linac.

## 2 MATERIALS AND METHODS

All the measurement data were collected on a preclinical MR‐Linac (Elekta AB) installed at Froedtert Hospital and Medical College of Wisconsin Cancer Center. The MR‐Linac is equipped with an electronic portal imaging device (EPID), which was used to localize dosimeters and phantoms spatially since there is no laser system currently installed in the MR‐Linac environment. The image pixel size for EPID is approximately 0.2 mm. The uncertainty of the alignment of the dosimeters and phantoms based on EPID is no more than 1 mm. The devices and measurement setups are described in details in Sections 3, 4, 1, 2 and 2.62.2.

The dosimetric data in the same conditions of the measurements were generated with the prototype Monaco TPS using the beam model provided by the vendor. For the end‐to‐end tests, a variety of dosimetric plans including three 3D static plans with single rectangular fields and two IMRT patient and QA plans were generated using the TPS and were delivered on the MR‐Linac. The details of the plans are described in Sections 2.52.1 and 2.62.2.

### 2.A Water tank measurements

Most relative and absolute dosimetry measurements were performed using an in‐house built water tank with a hand‐cranked gear mechanism to position the dosimetry detector at a required depth with increments of 0.1 mm. The volume of the water tank was 52.3 cm (X) x 42 cm (Y) x 24 cm (Z), where the X direction is oriented from patient left to right; Y is oriented patient inferior to superior; and Z is oriented from patient anterior to posterior. The hand‐cranked gear can slide in x direction with an attached ruler to adjust the X position for cross‐plan beam profile measurements. The table coordinates were used to determine the Y positions for in‐plan beam profile measurement. The distance from water surface to the couch top was 26.6 cm and the distance from the couch top to the machine isocenter is 14.3 cm, which results in an SSD of 131.2 cm since the SAD of the MR‐Linac is 143.5 cm.

The detectors used for water tank measurements include: PTW TN 30013 Farmer ion chamber (IC) (S/N: 9003; nominal sensitive volume is 0.6 cm^3^); PTW FREIBEUG N31014 micro IC (previously N31006, S/N: 0177; nominal sensitive volume is 0.015 cm^3^) and PTW TN 60019 microdiamond detector (S/N: 122257, active volume: 0.004 mm^3^). For the Farmer IC measurement, the long axis of the chamber was oriented along the longitudinal direction of the couch and parallel to the direction of the magnetic field. The measurements with micro chamber and diamond detector were obtained with the long axis of the detector parallel to the beam direction at gantry 0°, thus perpendicular to the magnetic field. The alignment of the center of the detector with the radiation center was confirmed with the portal imager at gantry angles 0°, 90°, 180°, and 270° and the positon uncertainty was less than 1.0 mm.

Considering the spatial limits between the isocenter and couch top and the geometry of the water tank, all percent depth dose (PDD) and beam profile measurements were performed at SSD = 131.2 cm, rather than 143.5 cm (the MR‐Linac isocenter). The total backscatter was 12.3 cm of water plus 1 cm of acrylic (bottom of the water tank) and the couch top when the detector located at the isocenter.

All measurements were performed with the gantry at 0° (i.e., perpendicular to the water surface). The field sizes and depth for all the PDD and beam profile measurements are listed in Tables 1 and 2. The field size is defined at the isocenter.

**Table 1:** Summary of the field size (FS) and depth for percent depth dose (PDD) measurements with three different types of detectors (Farmer ion chambers (IC), Diamond detector and Micro IC).

PDD	Detectors for water tank measurement		
	Farmer IC	Diamond	Micro IC
FS (cm^2^)	5 × 5, 10 × 10, 15 × 15, 20 × 20, 30 × 22	1 × 1, 2 × 2, 3 × 3, 5 × 5, 10 × 10	1 × 1, 2 × 2, 3 × 3, 5 × 5, 10 × 10

**Table 2:** Summary of the beam profile measurements with different dosimeters. (FS: Field size).

Profile		Detector for water tank measurement			Radiochromic film	IC profiler
		Farmer IC	Diamond	Micro IC		
In‐plane	FS (cm^2^)	—	—	1 × 1, 2 × 2, 3 × 3, 5 × 5, 10 × 10	1 × 1, 2 × 2, 3 × 3, 5 × 5, 10 × 10	5 × 5, 10 × 10, 15 × 15, 20 × 20, 30 × 22
	Depth (cm)	—	—	1.5, 10.0	1.5	1 × 1, 2 × 2, 3 × 3, 5 × 5, 10 × 10
Cross‐plane	FS (cm^2^)	5 × 5, 10 × 10, 15 × 15, 20 × 20, 30 × 22	1 × 1, 2 × 2, 3 × 3, 5 × 5, 10 × 10	1 × 1, 2 × 2, 3 × 3, 5 × 5, 10 × 10	1 × 1, 2 × 2, 3 × 3, 5 × 5, 10 × 10	5 × 5, 10 × 10, 15 × 15, 20 × 20, 30 × 22
	Depth (cm)	1.5, 5.0, 10.0, 15.0	1.5, 5.0, 10.0, 15.0	1.5	1.5	1 × 1, 2 × 2, 3 × 3, 5 × 5, 10 × 10

### 2.B Radiochromic film measurements

Radiochromic film (GAFchromic EBT‐3) was used as a secondary validation of measurements obtained from other detectors, particularly for small field sizes, for example, in the range of 1 × 1 to 5 × 5 cm^2^. The EBT3 film was sandwiched between slabs of solid water and positioned on the couch so that the film was oriented coronally at depth 1.5 cm with SSD 131.2 cm. The cross‐plane beam profiles were extracted for field size 1 × 1, 2 × 2, 3 × 3, and 5 × 5 cm^2^ using a film processing software (Radiological Imaging Technology, Inc).

### 2.C MR‐Compatible 2D ion chamber array measurements

In‐plane and cross‐plane beam profiles were also measured using an MR‐compatible 2D ion chamber array (IC profiler, Sun Nuclear Corporation, Melbourne, FL) for field sizes 5 × 5, 10 × 10, 15 × 15, 20 × 20, and 30 × 22 cm^2^ with gantry 0° at depths of 1.5, 5.0, 10.0, and 15.0 cm. The SSD was 131.2 cm, which is the same as the water tank measurements for the convenience of comparison. There was a 1 cm inherent water equivalent build up within the IC profiler itself.

### 2.D Reference dose verification

Reference dose verification was performed in liquid water using an ion chamber based on O'Brien et al.'s formalism^18^ for reference dosimetry in magnetic fields. Given the difficulties to setup the standard TG‐51^30^ reference condition with the MR‐Linac geometry, the formalism of Alfonso et al^31^ for small and nonstandard beams was adopted and corrected for the purpose of reference dose verification. In Alfonso's formalism, a set of machine specific‐reference (msr) conditions (e.g., smaller field size, different phantom shape, and material) were introduced. The absolute dose to water in an applied magnetic field B for the machine‐specific reference field size f_msr_ can be determined using Eq. (1)^18^:

(1) $$D_{w,Q_{\mathrm{msr}}}^{B,f_{\mathrm{msr}}}=M_{Q_{\mathrm{msr}}}^{B,f_{\mathrm{msr}}}N_{D,w,Q_{0}}k_{Q_{\mathrm{msr}}}^{B,f_{\mathrm{msr}}},\;\mathrm{where}\;k_{Q_{\mathrm{msr}}}^{B,f_{\mathrm{msr}}}=k_{Q,Q_{0}}k_{Q_{\mathrm{msr}},Q}^{f_{\mathrm{msr},}f_{\mathrm{ref}}}k_{B}^{Q_{\mathrm{msr}}}$$

where $k_{B}^{Q_{\mathrm{msr}}}$ is a correction factor to account for the magnetic field effect on the dose response of the ion chamber. For our reference dose verification, k_Q, Q0_ was determined based on TPR_20,10 _measurement according to the TRS‐398 code –of‐practice.^32^ The field size of 10 × 10 cm^2^ (defined at isocenter) was used for the calibration. Thus:

(2) $$k_{Q_{\mathrm{msr}},Q}^{f_{\mathrm{msr}},f_{\mathrm{ref}}}=1.$$

The $k_{B}^{Q_{\mathrm{msr}}}$ was chosen with reference to Table 3 of O'Brien et al.'s formalism.^18^ The measurement was performed at SSD = 133.5 cm and the ion chamber was placed at a depth of 10 cm. The tissue maximum ratio (TMR) at depth 10 cm was also measured to convert the dose at the point of measurement to the reference point which was chosen to be d_max_ at SAD to mimic the other Linacs in our clinic. An ADCL‐calibrated local standard PTW 30013 water‐proof Farmer chamber and Keithley electrometer (#35614) were used for the measurement.

**Table 3:** The comparison of the depth of the maximum percent depth dose for different field size between the calculation from the treatment planning systems and measurements with different detectors [Farmer ion chambers (IC), Diamond detector, and the micro IC].

Field size (cm^2^)	D_max_ (cm)			
	Beam model	Farmer IC	Diamond	Micro IC
10 × 10	1.4	1.4	1.4	1.4
5 × 5	1.4	1.4	1.5	1.4
3 × 3	1.3		1.3	1.2
2 × 2	1.1		1.1	1.2
1 × 1	1.0		1.1	1.0

### 2.E Data calculated from beam models

The dosimetric data in the same conditions as in the measurements described above were generated using the prototype Monaco TPS with the existing MR‐Linac beam model (7.0FFF + cryostat) using a calculation grid of 3 mm × 3 mm × 3 mm. The data were extracted from 3D plans of single beams irradiating onto a rectangular water phantom similar to the water tank. The PDDs along the CAX and the beam profiles for each field size at corresponding depths were extracted from the corresponding 3D dose distributions.

### 2.F End‐to‐end tests with an MR compatible 3D diode array

For the end‐to‐end tests, three single‐beam 3D plans based on CT data of an MR‐compatible cylindrical 3D diode array (ArcCheck, Model 1220‐MR, Sun Nuclear Corporation, Melbourne, FL) were generated for a rectangular field of 5 × 10 cm^2^ at gantry angles 0°, 90°, and 270°, respectively. In addition, two realistic step‐and‐shoot IMRT plans were generated based on two sample patient CT sets of pancreas cancer using commonly used dose‐volume constraints. The corresponding QA plans for the IMRT plans were created based on the ArcCheck CT set following the clinical procedure. The 3D plans and IMRT QA plans were generated on ArcCheck such that the position of the isocenter in the TPS matched the isocenter of the MR‐Linac. All these plans were delivered to the ArcCheck on the MR‐Linac following the workflow built in the MR‐Linac. The ArcCheck was aligned manually by comparing a serial of measured dose maps of a 5 × 10 cm^2^ beam at gantry angles of 0°, 90°, and 270° with the corresponding dose distributions generated with the TPS. The dose calibration for the ArcCheck was performed with MR‐Linac and the Diode array calibration was performed using a 6 MV, 10 × 10 cm^2^ photon beam from a conventional linac (Versa‐HD, Elekta AB, Stockholm, Sweden). The measured dose maps from the diode array of ArcCheck were compared with the dose distributions from the TPS with a commonly used gamma analysis (3%/3 mm with 5% threshold).

### 2.G Dose verification in inhomogeneous phantom

To verify the TPS dose calculation accuracy in inhomogeneous tissue, we performed dose measurements in a homemade phantom using an MR‐compatible ion chamber (A26MR, Standard Imaging) with a sensitive volume of 0.016 cm^3^. The inhomogeneous phantom was composited of nine slabs of different relative electron densities (rED) similar to lung, soft tissue, and bones. The dose distribution of a single beam with 10 × 10 cm^2^ field size and 200 MU at gantry 0° from the MR‐Linac was calculated in the TPS on the CT of the phantom in SAD setup. The calculated mean dose in the sensitive volume of the ion chamber located at the depth of 11 and 0.3 cm off central axis in the phantom was extracted from the TPS and was compared with the measurement.

## 3 DATA ANALYSIS AND RESULTS

### 3.A Percentage depth dose

**Figure 1:**
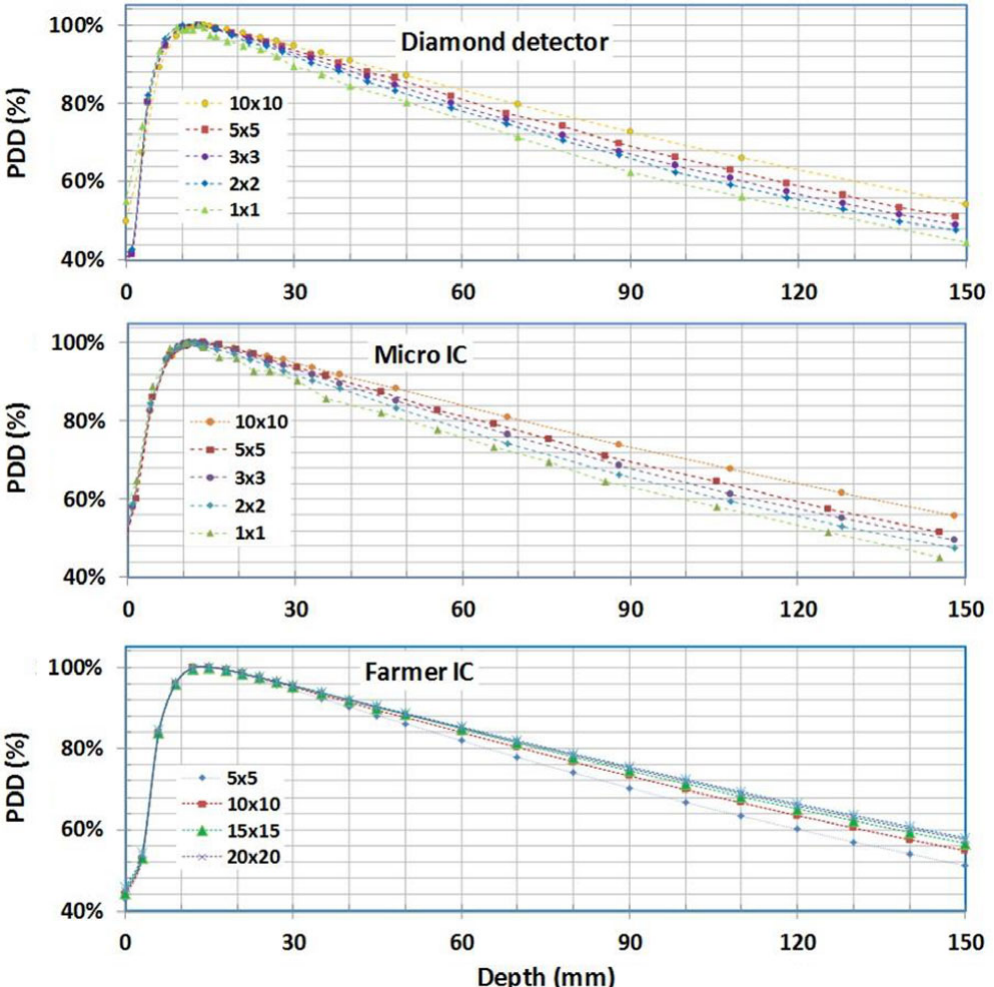
Percentage depth dose curves measured in liquid water using micro ion chambers (IC), diamond detector, and Farmer IC.

Figure 1 displays PDDs measured with Farmer IC, diamond detector, and micro IC. Since the PTW Farmer ion chamber has the largest effective detector volume, it was used for dosimetry measurements of the larger field sizes (≥5 × 5 cm^2^). With much smaller sensitive volume, the diamond detector and micro IC were used for small field measurements (<5 × 5 cm^2^). To check the consistency between the various detectors, the PDD for field sizes of 5 × 5 and 10 × 10 cm^2^ were also measured with both diamond detector and micro IC.

**Figure 2:**
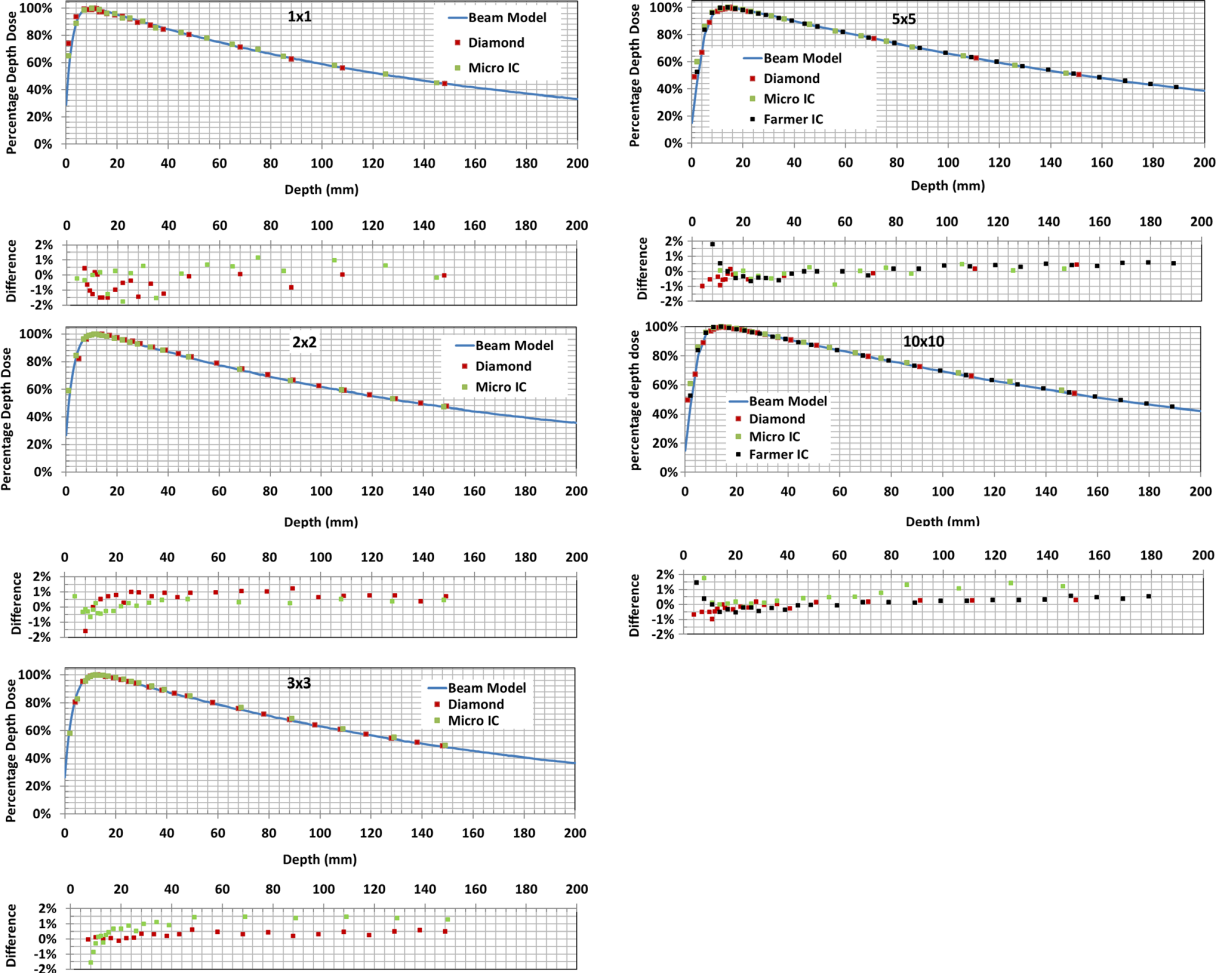
Measured and calculated percent depth doses (PDDs) for different field sizes. Differences between measured and calculated PDD are plotted below each PDD.

The measured PDDs were compared against those calculated from the beam model in the TPS for field sizes ≤ 10 × 10 cm^2 ^in Fig. 2. The differences between the measured and calculated PDDs are also shown in Fig. 2. Beyond the buildup region, the PDDs measured with all detectors agreed well with the beam model. The absolute difference for most measurement points was < 1% (range ±2%). In general, the measurements with the diamond detector lead to smaller deviations from those by the beam models as compared to those by the micro IC. The depth of maximum (i.e., d_max_) decreases with decreasing field size, from approximately 1.4 cm for field size 10 × 10 cm^2^ to 1.0 cm for field size 1 × 1 cm^2^, as shown in both measured and calculated data in Table 3.

### 3.B Cross‐plane and in‐plane beam profiles

**Figure 3:**
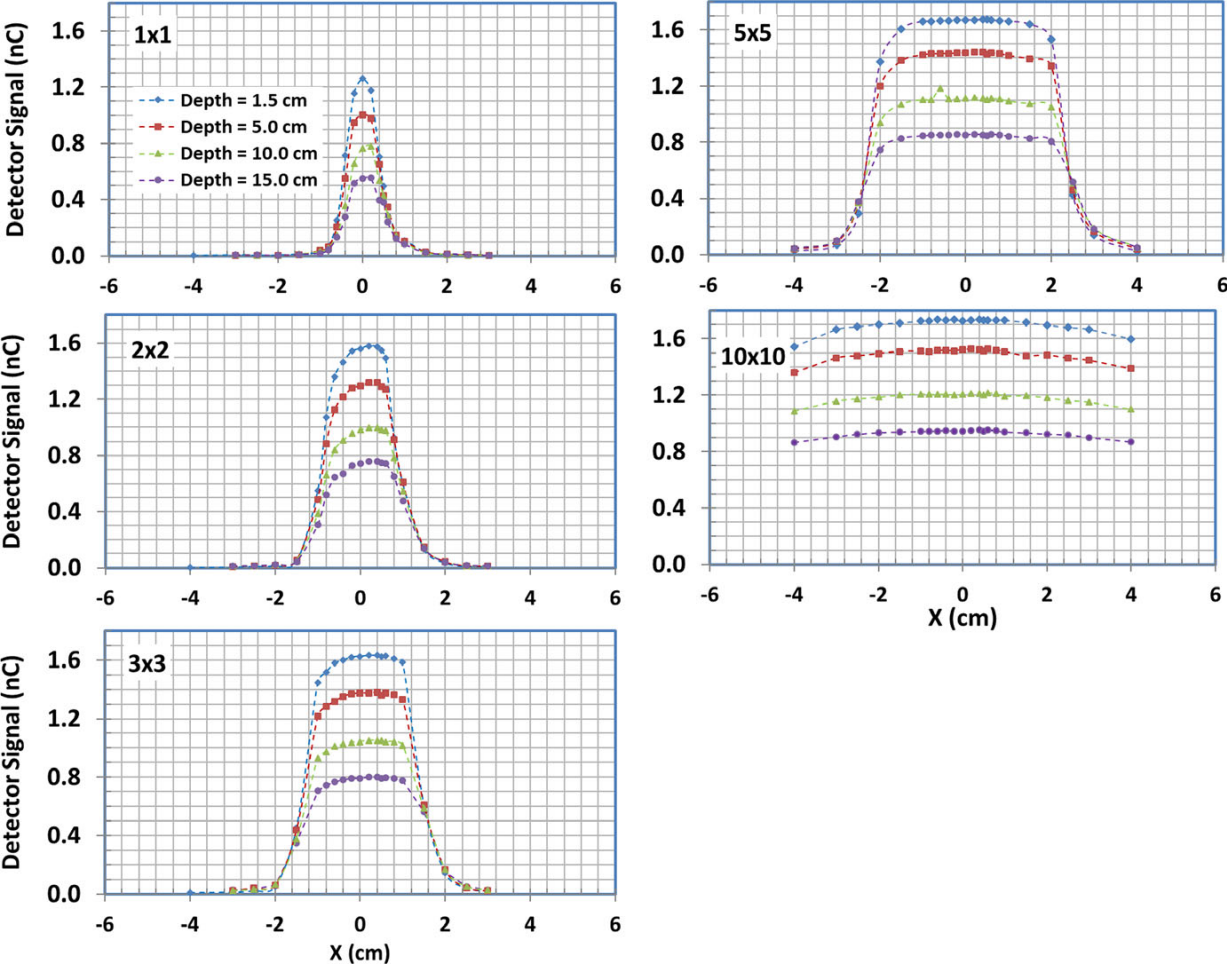
Cross‐plane beam profiles measured with diamond detector for different field sizes and depths in liquid water.

**Figure 4:**
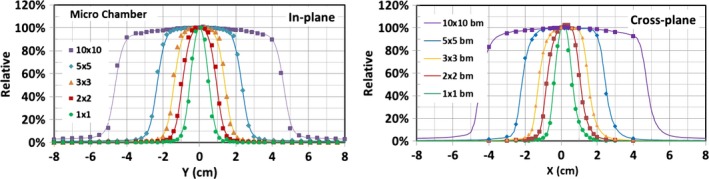
In‐plane and cross‐plane beam profiles measured with micro ion chambers for different field sizes at depth of 1.5 cm along with the beam model calculations.

Since the magnetic field affects dosimetry mostly in transverse planes, the majority of the data were collected in the cross‐plane direction. The large field cross‐plane beam profiles (≥5 × 5 cm^2^) were measured with the Farmer IC and IC profiler^TM^. Those for small field sizes, for example, 1 × 1, 2 × 2, 3 × 3, and 5 × 5 cm^2^, were measured with diamond detector, micro IC, and radiochromic film. For the consistency check, the cross‐plane beam profiles for field size 10 × 10 cm^2^ was also measured with both the diamond detector and micro IC. Due to the geometrical limitations of the water tank for larger field sizes (≥10 × 10 cm^2^), only a portion of the cross‐plane beam profiles could be obtained. Figure 3 displays cross‐plane beam profiles measured with the diamond detector at different depths (1.5, 5.0, 10.0, and 15.0 cm) for different field sizes. The deviations of the center of the cross‐plane profiles from the CAX were extracted and compared to the beam model calculation for different depth and field size as shown in Table 4. The in‐plane beam profiles for field sizes: 1 × 1, 2 × 2, 3 × 3, 5 × 5, and 10 × 10 cm^2 ^were measured with the micro IC. These in‐plane measurements at depth 1.5 cm along with the cross‐plane profiles measured with the same chamber are shown in Fig. 4.

**Table 4:** The deviations (unit: cm) of the center of the cross‐plane profiles from the central axis for different field sizes (1 × 1, 2 × 2, 3 × 3, 5 × 5 and 10 × 10 cm^2^) at different depth (1.5, 5.0, 10.0 and 15.0 cm). (Meas.: Measurement; B.M.: Beam Model).

Field size/depth (cm)	1 × 1		2 × 2		3 × 3		5 × 5		10 × 10	
	Meas.	B.M.	Meas.	B.M.	Meas.	B.M.	Meas.	B.M.	Meas.	B.M.
1.50	0.10	0.11	0.09	0.13	0.14	0.13	0.13	0.14	–	0.14
5.00	0.12	0.12	0.13	0.14	0.15	0.14	0.13	0.15	–	0.15
10.00	0.17	0.13	0.17	0.15	0.20	0.15	0.16	0.16	–	0.16
15.00	0.19	0.14	0.20	0.16	0.24	0.16	0.20	0.17	–	0.17

**Figure 5:**
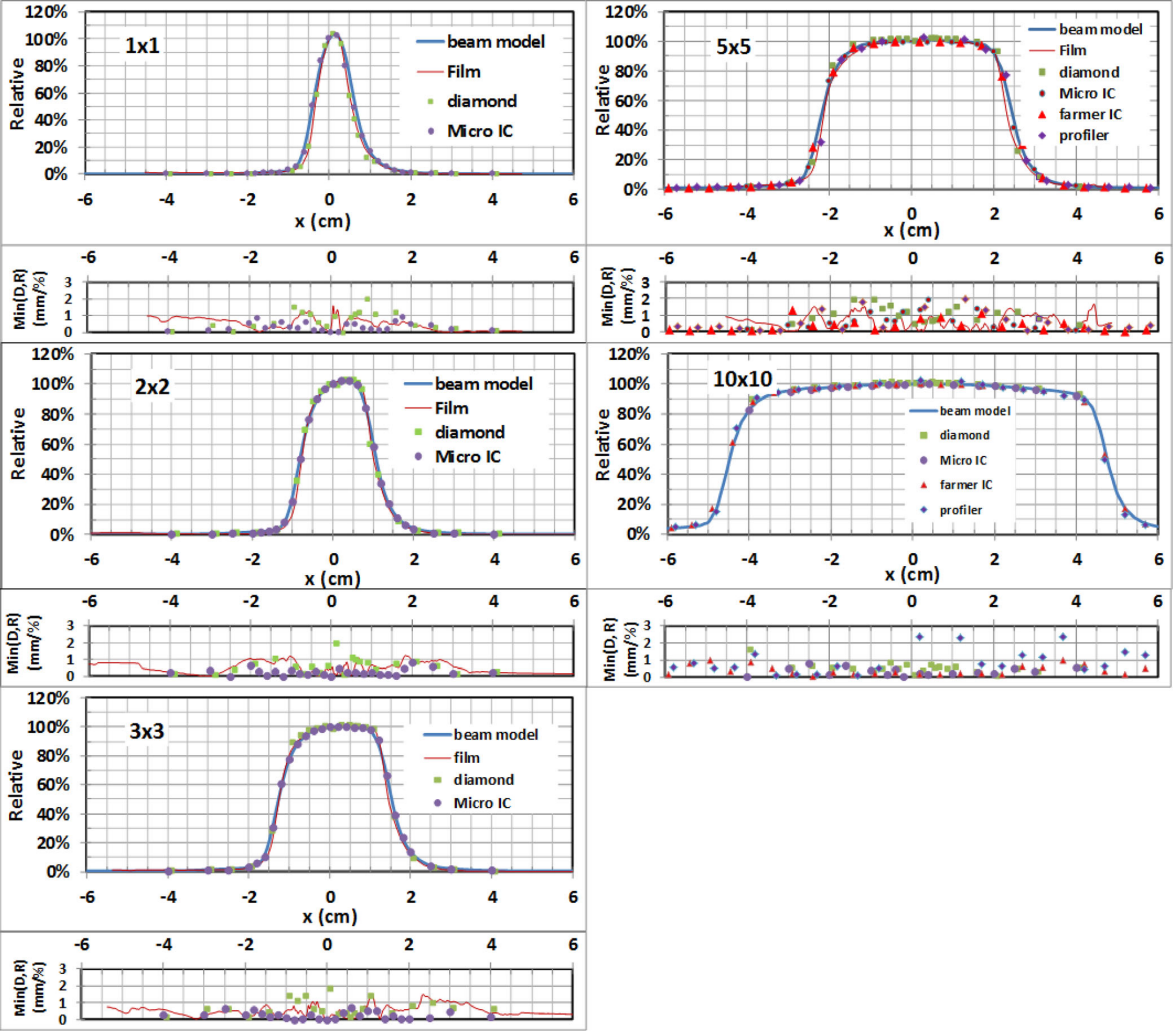
Measured and modeled cross‐plane beam profiles across multiple field sizes at depth = 1.5 cm. Min(D/R)(mm/%): The minimum of the distance (mm) difference and relative dose (%) difference between measurements and beam model.

Cross‐plane beam‐profile measurements at depth 1.5 cm with different detectors for field sizes 1 × 1, 2 × 2, 3 × 3, 5 × 5, and 10 × 10 cm^2^ are compared with the beam model calculations in Fig. 5. The minimum of the distance (mm) difference and relative dose (%) difference between measured and modeled profiles are plotted in the same figure. In general, the beam profiles measured with different detectors agree well with those from the beam models, with no more than 1% difference in the regions outside the penumbra and no more than 2% for most regions within the primary beam, except within the penumbra regions. In the penumbra regions, the agreement is within 1.0 mm for most data points of all field sizes studied. The film and diamond detector measurements generally display sharper falloff in penumbra regions compared to those from the beam model and micro IC measurements. The beam model correctly models ERE by using tilted dose kernels. It is clear that there are lateral shifts (i.e., asymmetry) of the cross‐plane beam profiles to the direction of patient‐left. The shift of the center of the cross‐plane profile increases slightly with the depth and field size, but the measured differences of the shift are within 1 mm compared to 0.5 mm calculated with the beam model (see Table 4). As expected, no lateral shifts in in‐plane beam profiles were observed and the dose distribution is symmetric and peaks at CAX for both measured and calculated data.

### 3.C Reference dose verification

Based on the type of ion chamber and its orientation with respect to the irradiation and magnetic fields, a $k_{B}^{Q_{\mathrm{msr}}}$ of 0.994 was used for the reference dose verification.^18^ The absolute dose at the measurement point was determined to be 87.66 cGy per 100 MU. A reference dose of 111.8 cGy/100 MU was obtained at the depth of maximum dose with SAD setup (d_max_, SAD) when a TMR of 0.784 was used to convert the dose at the measurement point to the reference point.

### 3.D End‐to‐end tests

**Figure 6:**
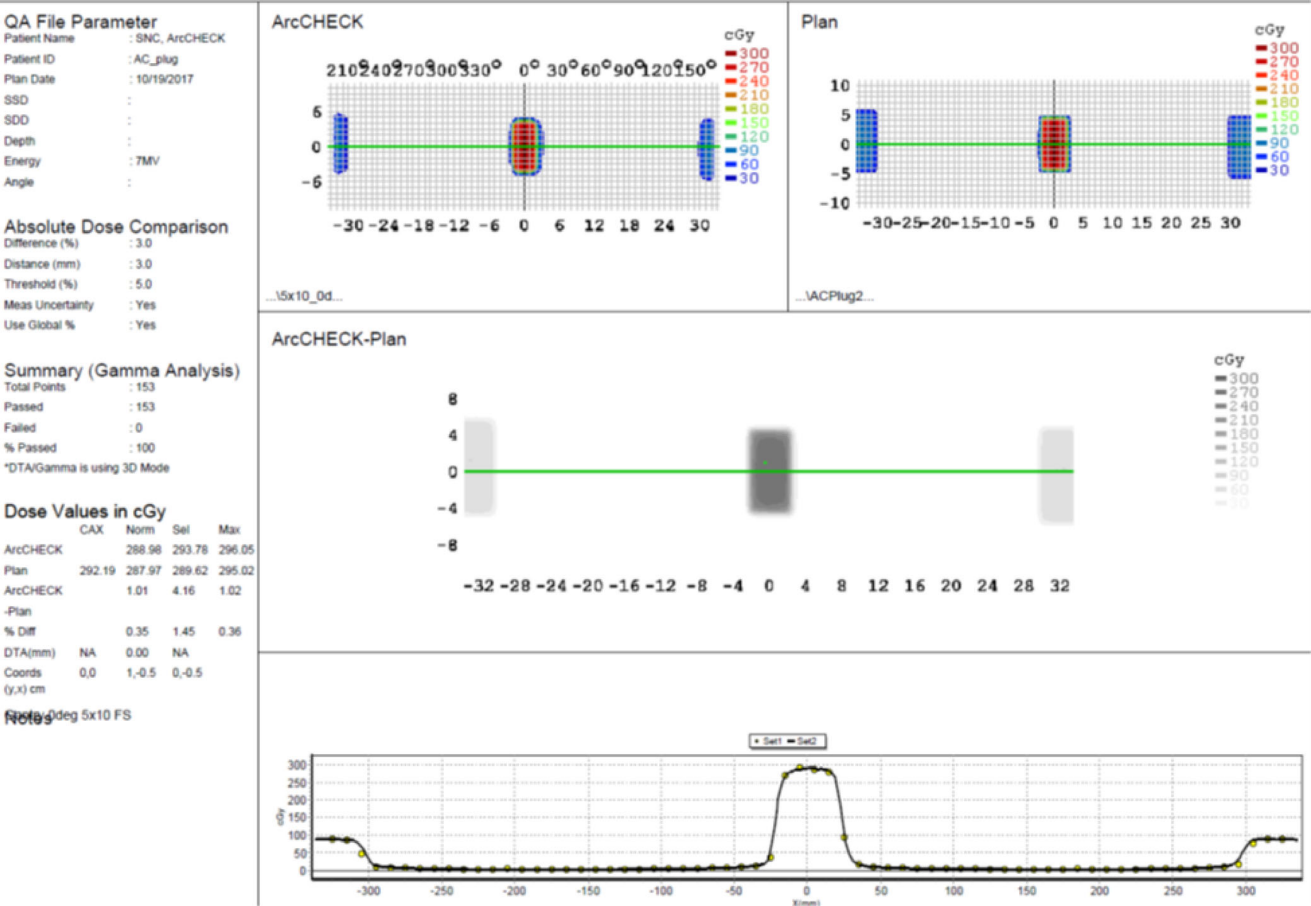
Dose comparison between planned and ArcCheck measured 5 × 10 cm^2^ rectangular field at gantry 0° using SNC Patient^TM^ software.

The three single‐beam plans with a 5 × 10 cm^2 ^ field at gantry 0°, 90°, and 270° were delivered to the ArcCheck and the measured dose was compared with the TPS calculations. Figure 6 displays dose comparison between a planned and ArcCheck measured 5 × 10 cm^2^ rectangular field at gantry 0° using SNC Patient^TM^ software. The test beams at three gantry angles all passed with more than 99% in absolute mode using gamma analysis and clinic criteria. These excellent agreements indicate that the ArcCheck was aligned properly.

**Figure 7:**
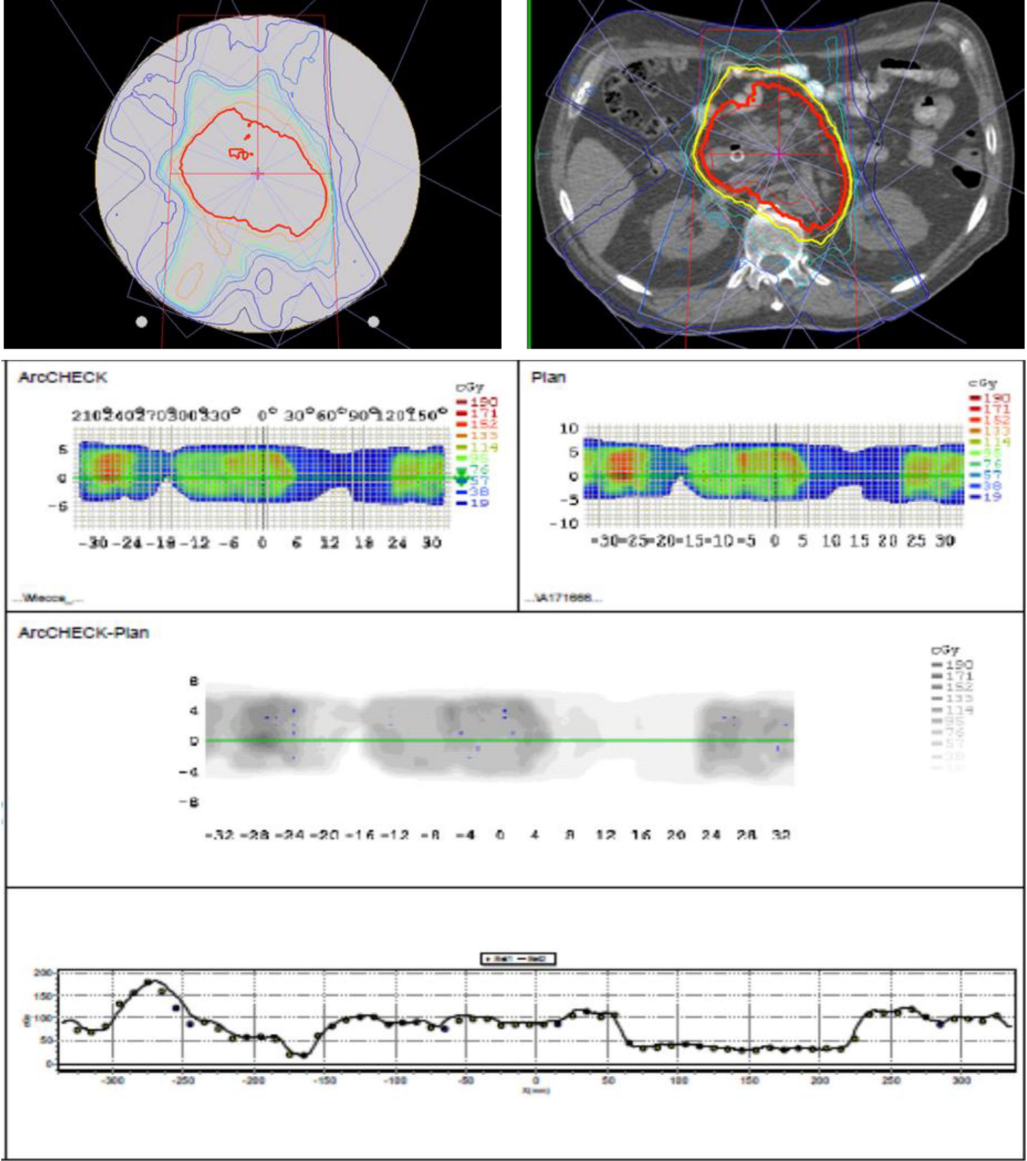
A comparison of measured and calculated dose maps for an IMRT plan. Top left: Dose distribution of the QA plan on ArcCheck CT; Top right: Dose distributions on the patient CT; Bottom: Comparisons of doses measured (ArcCheck) and calculated (Plan) as analyzed with SNC Patient^TM^.

The two step and shoot IMRT QA plans, with 6 and 5 beam angles, respectively, were delivered to the ArcCheck on MR‐Linac. The workflow for the IMRT delivery was: The IMRT plan generated with TPS is exported to the Record‐and‐Verify system (MOSAIQ, Elekta AB) and is then imported into Treatment Service Manager (TSM); the TSM communicates with the MR‐Linac console and controls the delivery of each beam to the ArcCheck. The measured dose maps were compared with the plan dose maps using SNC Patient^TM^ software and the QA plans were successfully passed with 96.5% and 97.7% using gamma analysis with clinical criteria. Figure 7 presents the analysis of comparisons between the measured and planed dose distributions for one of the IMRT plan, along with the isodose lines of the patient and QA plans on patient and ArcCheck CTs.

### 3.E Dose verification in inhomogeneous phantom

**Figure 8:**
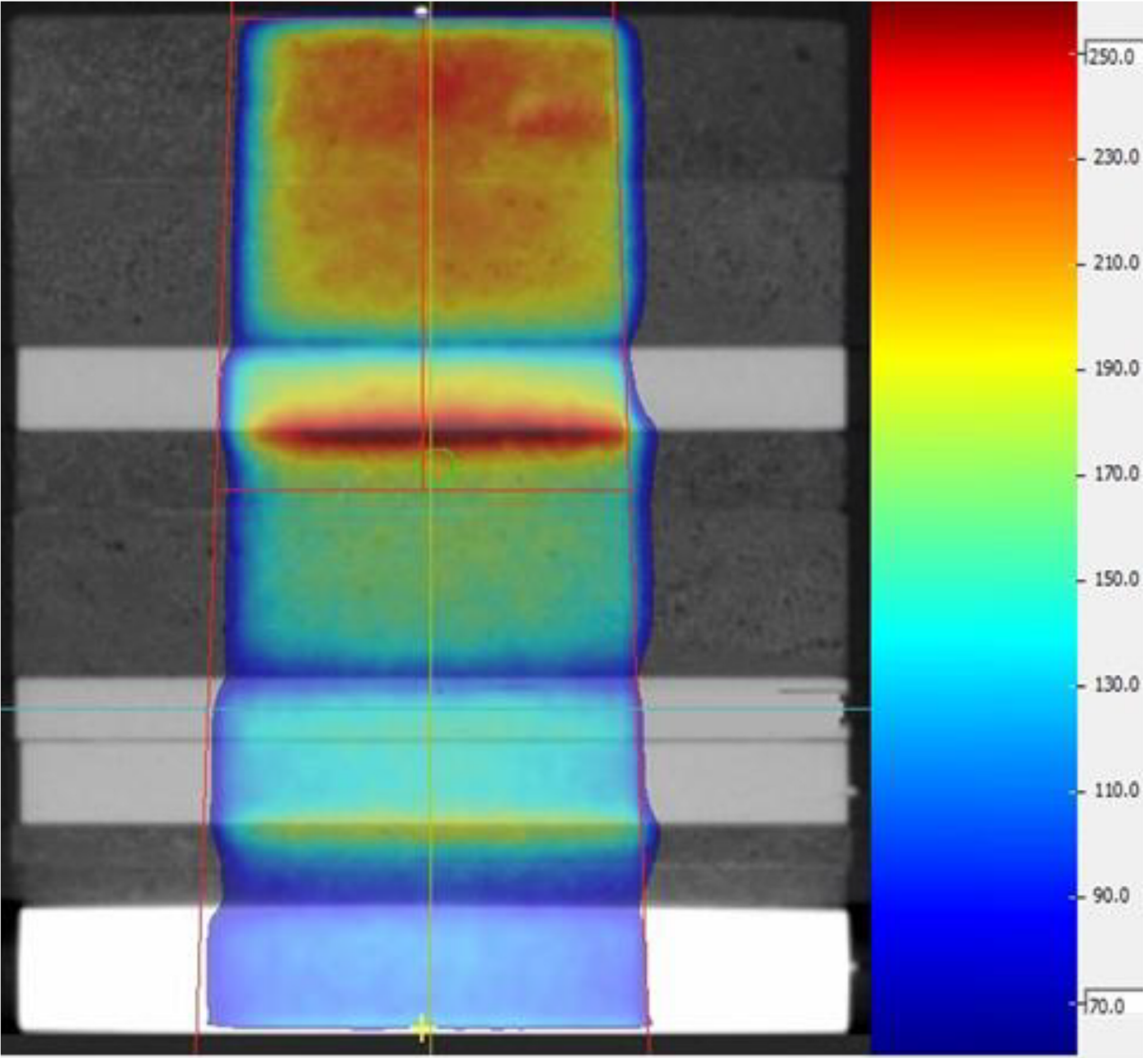
Single beam dose distribution on a transverse plane view of the inhomogeneous phantom. The green circle indicates the location of the ion chamber.

Figure 8 shows the TPS calculated dose distribution on a transverse plane of the inhomogeneous phantom. The ERE is obvious at the interfaces from higher rED to lower rED materials in the beam path. The dose measured with ion chamber on the MR‐Linac after daily output correction was 0.98% different from the calculated mean dose in the chamber sensitive volume.

## 4 DISCUSSION AND CONCLUSIONS

The beam model to account for ERE for a high‐field MR‐Linac was validated with measurements of relative and absolute dosimetry. The ERE is visible in penumbra especially for small fields which is correctly modeled by the TPS. Small dosimeter such as the micro ion chamber and the diamond detector used in this work is found to be acceptable for small field dosimetry for the MR‐Linac. The diode array, such as the IC profiler, may be used for more frequent beam profile QA purposes for field sizes ≥ 5 × 5 cm^2^, while radiochromic film can be used for frequent small field size < 5 × 5 cm^2 ^beam profile checks.

Compared to the photon beam of similar energy on a conventional Linac, the depth of the maximum dose is reduced due to the presence of the magnetic field. The maximum intensity of cross‐plane beam profile shifts to the patient left, perpendicular to the magnetic field, for small fields. The cross‐plane profiles become asymmetric due to the titled dose kernel arising from the magnetic field. The shift increases slightly with the depth and the field size (≤0.5 mm from beam model calculation), while the in‐plane profiles remain symmetric as those without a magnetic field, since the magnetic field runs parallel in this orientation.

The absolute dosimetry measurements include the following: (a) reference dose verification (i.e., reference dosimetry) based on the modified TG‐51 protocol considering the presence of the magnetic field; (b) the end‐to‐end tests for several simple plans of 5 × 10 cm^2^ rectangular fields at different gantry angles; (c) the end‐to‐end tests of two IMRT plans generated for MR‐Linac, and (d) the dose verification in inhomogeneous phantom. The measurements performed during the deliveries of these plans on the MR‐linac passed absolute dosimetric criteria commonly used in the clinic.

All the beam PDD and profile measurements in this study were performed on a preclinic MR‐Linac. Although there is no difference between this device and its clinical version (Unity, Elekta Inc) in their beam generation and shaping systems, a major portion of the reported verification measurements should be repeated on a clinical MR‐Linac before it is used for patient.

Both the relative and absolute dosimetric measurements on the MR‐Linac indicate that the prototype Monaco TPS and beam model accurately account for the effects of the 1.5 T, transverse magnetic field. The end‐to‐end tests verified the entire treatment planning and delivery process.

## CONFLICTS OF INTEREST

The authors declare there is no conflict of interest.
